# The analysis of translation-related gene set boosts debates around origin and evolution of mimiviruses

**DOI:** 10.1371/journal.pgen.1006532

**Published:** 2017-02-16

**Authors:** Jônatas Santos Abrahão, Rodrigo Araújo, Philippe Colson, Bernard La Scola

**Affiliations:** 1 Unité de Recherche sur les Maladies Infectieuses et Tropicales Emergentes (URMITE) UM63 CNRS 7278 IRD 198 INSERM U1095, Aix-Marseille Univ., 27 boulevard Jean Moulin, Faculté de Médecine, Marseille, France; 2 Instituto de Ciências Biológicas, Departamento de Microbiologia, Laboratório de Vírus, Universidade Federal de Minas Gerais, Belo Horizonte, Brazil; CNRS, FRANCE

## Abstract

The giant mimiviruses challenged the well-established concept of viruses, blurring the roots of the tree of life, mainly due to their genetic content. Along with other nucleo-cytoplasmic large DNA viruses, they compose a new proposed order—named *Megavirales*—whose origin and evolution generate heated debate in the scientific community. The presence of an arsenal of genes not widespread in the virosphere related to important steps of the translational process, including transfer RNAs, aminoacyl-tRNA synthetases, and translation factors for peptide synthesis, constitutes an important element of this debate. In this review, we highlight the main findings to date about the translational machinery of the mimiviruses and compare their distribution along the distinct members of the family *Mimiviridae*. Furthermore, we discuss how the presence and/or absence of the translation-related genes among mimiviruses raises important insights to boost the debate on their origin and evolutionary history.

## Introduction

In 1957, a time when genetics and cellular biology were in their preliminary stages, André Lwoff proposed a modern concept of viruses based on a set of features that, directly or indirectly, emphasized that viruses are defined by “negative plesiomorphic or apomorphic non-natural characteristics” [1]. In the following years, many research fields evolved—including molecular biology and virology—and several different viruses were discovered, presenting some characteristics which had never been seen before among living organisms. However, even with such progress, most of the features raised by Lwoff have still been well supported if we consider the universe of viral species catalogued by the International Committee on Taxonomy of Viruses (ICTV) [2].

However, the discovery of the giant viruses blurred some of those well-established concepts, surprising the scientific community by their size and genetic content [3]. Although they still fit in some of Lwoff’s proposed non-natural features (the same is also true for some intracellular bacteria), giant viruses present an astonishing arsenal of genes not widespread in the virosphere, some of them related to important steps of the translational process, including transfer RNAs (tRNAs), aminoacyl–tRNA synthetases (aaRS), and translation factors for peptide synthesis [4–6]. Recent studies have shown that some of those genes can be related to the improvement of viral fitness, despite the presence of related genes in their hosts’ genomes [7]. The analysis of these intriguing translation-related gene sets has raised interesting theories about the “lifestyle” of giant viruses’ ancestors. In this review, we highlight the main features about the diversity, function, and putative origin of mimivirus translation-related genes.

### *Mimiviridae*: A giant virus family with exceptional genetic content

The first giant virus was isolated from a water sample of a cooling tower in Bradford, England, replicating in the protist host *Acanthamoeba polyphaga*. At the time of its isolation, the new microorganism was considered to be a gram-positive bacterium, and its viral nature was only established after transmission electron microscopy analysis, which led to its label *Acanthamoeba polyphaga mimivirus* (APMV) [8]. Since then, new mimivirus-like viruses have been isolated in different parts of the world. These viruses constitute the new family *Mimiviridae*, which was included in the recently proposed order *Megavirales* [9,10].

The mimiviruses present several unusual features, both genetic and structural (for details about the viral structure, see [11]). The genome of these viruses consists of a single linear dsDNA molecule, is A+T rich (reaching up to 1,259 Kb), and may have approximately 1,000 open reading frames with a coding density higher than 90% [4,12]. The mimiviruses are phylogenetically divided into two groups (I and II), the first, which comprises mimiviruses that infect *Acanthamoeba*, being subdivided into three lineages (A, B, and C). Lineage A comprises APMV [8], Mamavirus [13], Samba virus [14], Niemeyer virus [15], and many others. Lineage B is mainly represented by Moumouvirus [16] and lineage C by Megavirus chilensis (MCV) [6], Courdo11 virus [17], and LBA111 virus [18]. Group II comprises smaller *Mimiviridae* members distantly related to APMV and is represented by *Cafeteria roenbergensis* virus (CroV) [5] and some algae viruses, including *Phaeocystis globosa* virus (PgV) [19] and Organic Lake Phycodnaviruses (OLV) [20].

The genome of mimiviruses is impressive not only for its size but also (and mainly) for its genetic content, presenting many genes which have never previously been described for other viruses. The mimiviruses possess many genes codifying DNA repair enzymes and are the first viruses to code for topoisomerase type IA [4]. Moreover, mimiviruses have their own glycosylation apparatus, presenting glycosyltransferases that are involved in the biosynthesis of glycans and post-translational protein modifications [21,22]. Furthermore, and even more impressive, is the presence of genes related to the protein synthesis, such as aaRS, tRNAs, and translation factors, which are present in different amounts in several representatives of the family *Mimiviridae* (Table 1). Other giant viruses, such as Marseillevirus [23], Pandoravirus [24], Faustovirus [25], and Mollivirus [26], also have some of these components, but in much less abundance compared to the mimiviruses.

**Table 1:** Giant viruses’ translation-related genes. Representative isolates of each group or family.

Group/Viruses	Aminoacyl-tRNA synthetase	tRNA	Translation Factors
**Mimivirus Lineage A**			
APMV	ArgRS, CysRS, MetRS, TyrRS	Leucine (3x), Histidine, Cysteine, Tryptophan	IF4A, IF4E, SUI1, eF-TU, eRF1
Mamavirus	ArgRS, CysRS, MetRS, TyrRS	Leucine (3x), Histidine, Cysteine, Tryptophan	IF4A, IF4E, SUI1, eF-TU, eRF1
Lentille	ArgRS, CysRS, MetRS, TyrRS	Leucine (3x), Histidine, Cysteine, Tryptophan	IF4A, IF4E, eF-TU, eRF1
Hirudovirus	ArgRS, CysRS, MetRS, TyrRS	Leucine (3x), Histidine, Cysteine, Tryptophan	IF4A, IF4E, SUI1, eF-TU, eRF1
SMBV	ArgRS, CysRS, MetRS, TyrRS	Leucine (3x), Histidine, Cysteine, Tryptophan	IF4A, IF4E, SUI1, eF-TU, eRF1
OYTV	ArgRS (2x), CysRS, MetRS, TyrRS	Leucine (3x), Histidine, Cysteine, Tryptophan	IF4A, IF4E, SUI1, eF-TU, eRF1
KROV	ArgRS, CysRS, MetRS, TyrRS	Leucine (3x), Histidine, Cysteine	IF4A, IF4E, eF-TU, eRF1
AMAV	CysRS, TyrRS	Leucine (3x), Histidine, Cysteine, Tryptophan	IF4A, IF4E, SUI1, eF-TU, eRF1
NYMV	ArgRS, CysRS (2x), MetRS (2x), TyrRS (2x)	Leucine (2x), Histidine, Cysteine	IF4A, IF4E, SUI1, eF-TU, eRF1
Terra2	ArgRS, CysRS, MetRS, TyrRS	Leucine (2x), Histidine, Cysteine, Tryptophan	IF4A, IF4E, SUI1, eF-TU, eRF1
Bombay	ArgRS, CysRS, MetRS, TyrRS	Leucine (3x), Histidine, Cysteine, Tryptophan	IF4A, IF4E, SUI1, eF-TU, eRF1
**Mimivirus Lineage B**			
APMOUV	ArgRS (4x), CysRS, IleRS, MetRS, TyrRS	Leucine, Histidine, Cysteine	IF4E, SUI1, eF-TU, eRF1
Goulette	CysRS, MetRS	Leucine (3x), Histidine, Cysteine	IF4E, SUI1, eF-TU, eRF1
Monve	ArgRS (2x), AsnRS, CysRS, IleRS (2x), MetRS, TyrRS	Leucine, Histidine, Cysteine	IF4A, IF4E (2x), SUI1, eRF1
**Mimivirus Lineage C**			
MCV	ArgRS, AsnRS, CysRS, IleRS, MetRS, TrpRS, TyrRS	Leucine (2x), Tryptophan	IF4A, IF4E, SUI1, eF-TU, eRF1
Terra1	ArgRS, CysRS, MetRS, TyrRS	Leucine, Tryptophan	IF4A, IF4E, SUI1, eF-TU, eRF1
LBA111	ArgRS, AsnRS, CysRS, IleRS, MetRS, TrpRS, TyrRS	Leucine (2x), Histidine, Cysteine, Tryptophan	IF4A, IF4E, SUI1, eF-TU, eRF1
Courdo7	IleRS, TyrRS	Leucine (3x), Tryptophan	IF4A (2x), IF4E, SUI1, eRF1
Courdo11	ArgRS, AsnRS (2x), CysRS, IleRS, MetRS, TrpRS, TyrRS	Leucine (3x), Histidine, Cysteine, Tryptophan	IF4A (2x), IF4E, SUI1, eRF1
**Mimivirus group II**			
CroV	IleRS	Leucine (9x), Serine (5x), Tyrosine, Asparagine, Lysine	IF4A, IF4E, SUI1
PgV	-	Leucine (3x), Asparagine (2x), Isoleucine, Arginine, Glutamine	IF4E
OLV	-	Leucine, Isoleucine, Tyrosine, Asparagine, Arginine	IF4E
**Other giant viruses**			
Marseillevirus	-	-	eIF5, SUI1, EF1α, eRF1
Faustovirus E12	-	-	SUI1
Pandoravirus salinus	TyrRS, TrpRS	Proline, Methionine, Tryptophan	IF4E
Pandoravirus dulcis	TyrRS	Proline	IF4E
Pandoravirus inopinatum	-	Proline	IF4E
Mollivirus sibericum	-	Leucine, Methionine, Tyrosine	IF4E

### Aminoacyl-tRNA synthetases among viruses—Breaking barriers

The aaRS are key enzymes in gene translation, during which they catalyse the esterification of a specific amino acid to the 3’-end of its cognate tRNA, forming the aminoacyl-tRNAs [27,28]. There are 20 different aaRS, which are divided into two families named class I and class II [28]. The aaRS are present in a wide variety of different organisms from all domains of life and, until very recently, there were no descriptions of these enzymes in a virus, and they were thus considered trademarks of cellular organisms [29]. However, with the discovery of the mimiviruses, this scenario has changed.

A total of four aaRS were found in the APMV genome (Arginyl-RS, Cysteinyl-RS, Methionyl-RS, and Tyrosyl-RS), all of them classified as class I aaRS [4]. In the following years, other mimiviruses were discovered, and the number of mimiviral aaRS expanded. Moumouvirus presents three out of four aaRS described in APMV (ArgRS, CysRS, and TyrRS), plus two others (Asparaginyl-RS [class II] and Isoleucyl-RS [class I]) [16]; and remarkably, MCV presents all the aaRS found in APMV and Moumouvirus, plus another one (Tryptophanyl-RS) [6]. Therefore, to our knowledge, MCV displays the most diverse set of aaRS, i.e., seven different aaRS. It is noteworthy that several paralogs of aaRS can be found in the genome of some mimiviruses. Moumouvirus has four copies of ArgRS and, for this reason, it is the virus with the highest abundance of aaRS (eight); and Niemeyer virus, a new lineage A mimivirus, presents three sets of aaRS duplication (MetRS, CysRS, and TyrRS are duplicated) [15,16]. Regarding CroV (mimivirus group II), only IleRS is present, which is also found in mimiviruses of lineages B and C [5].

**Fig 1:**
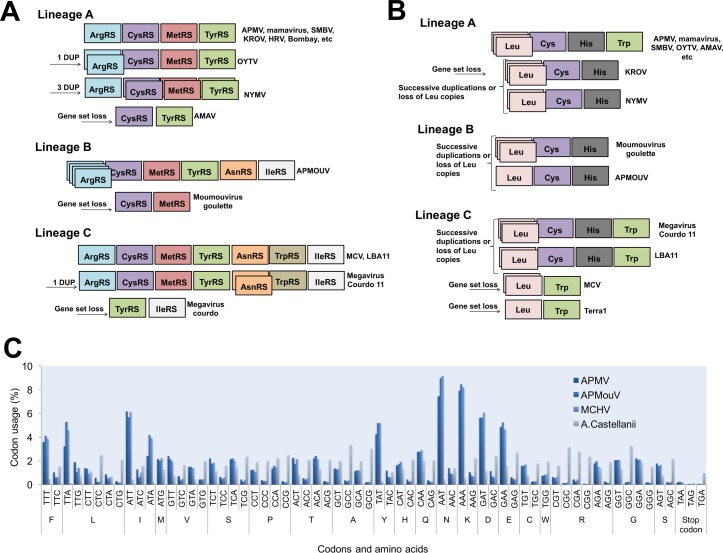
**Schematic view of mimiviruses’ aaRS (A) and tRNA (B) and codon/amino acid usage (C)**. The distribution of these genes follows a clear pattern of diversity among *Mimiviridae*: viral genomes with a wealthy aaRS and tRNA gene set (e.g., some lineage C isolates) contain all types of aaRS/tRNAs found in the rest of the family. This indicates a gradual inter- or intra-lineages gene loss throughout the evolution of different mimiviruses. The codon and amino acid usage analysis shows a clear difference between mimiviruses and *Acanthamoeba castellanii* patterns.

**Fig 2:**
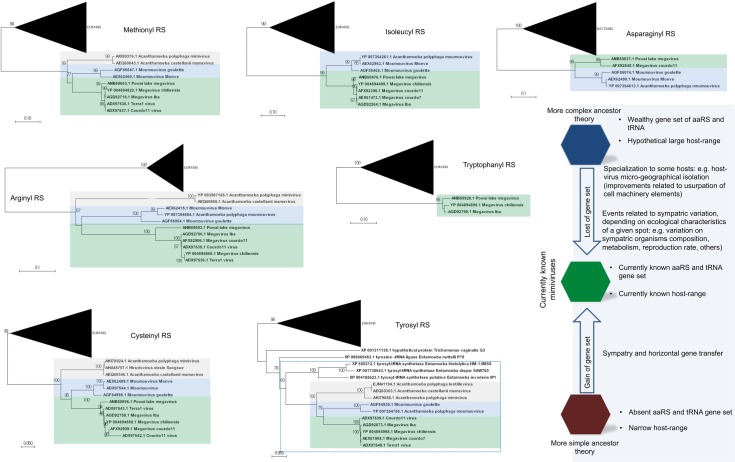
Phylogeny reconstruction of mimiviruses’ aaRS. The unrooted trees were generated using MEGA 7 with the maximum likelihood method based on all aaRS found in mimiviruses. The trees were obtained after the alignment of the 100 best hits found in Genbank after BLASTing Megavirus chilensis aaRS predicted aa against all databases. Bacteria or Eukarya taxon, when present, were condensed from the outermost branch presenting bootstrap value >90. Mimiviruses of lineages A (grey), B (blue), and C (green) are highlighted. In all trees but the TyrRS tree, mimiviruses do not cluster inside cellular organism’s branches. The bottom–right shows some evolutionary scenarios related to mimivirus evolution that considers their hosts and translation-related genetic data set.

The distribution of aaRS follows a clear pattern of diversity in family *Mimiviridae*: viral genomes with a wealthy aaRS gene set (e.g., MCV) contain all types of aaRS found in the rest of the family [6,13,15,16]. This suggests gradual gene loss throughout the evolution of different mimivirus groups (Fig 1A). The evolutionary pressures related to the conservation or loss of aaRS might be linked to specific environmental pressures to which each mimivirus group and/or lineage was submitted after mimiviral species radiation. This scenario would be in accordance with previous phylogenetic and phylogenomic works, which suggested that giant viruses originated from a more complex organism and evolved by genomic reduction [30,31]. However, other studies suggested that some mimivirus aaRS were acquired by horizontal gene transfer (HGT) [32,33]. From this perspective, the mimiviruses should have originated from smaller organisms and evolved mainly by HGT events, being considered “gene pickpockets” [32,33,61] (Fig 2). It is important to highlight, however, that the phylogeny of aaRS is quite complex and sometimes violates the expected pattern of canonical domains of life [29]. The methods of tree construction, alignment, and hits sampling could explain conflicting results observed in different studies (S1–S7 Files). Nevertheless, in Fig 2, we present eight mimivirus aaRS-based trees constructed by maximum likelihood method (very similar results were observed for trees constructed by the neighbor joining method). Considering about 100 of the best hits obtained in GenBank related to each aaRS, we observed that phylogenic reconstructions suggested that all mimiviral genes but TyrRS clustered together as an independent group, with bootstrap values >90 both for viral and cellular taxa in most of the trees (Fig 2). However, both theories regarding mimivirus origin—that mimiviruses either originated from a more complex ancestor or that they originated from a simple ancestor—are plausible, although increasing evidence points to the former hypothesis [4,10,30,31,34].

The analysis of APMV transcriptome revealed that its four aaRS are expressed during the replication cycle of the virus [35]. Furthermore, experimental data have demonstrated that some mimiviruses’ aaRS are indeed functional [4,36,37]. Protein structural and functional studies confirmed that APMV, MetRS, and TyrRS act as genuine enzymes. It was shown that TyrRS is a homodimer similar to other class I aaRS described so far, but in contrast to what is found in cellular organisms, the viral TyrRS seems to recognize only two bases in tRNA [37]. Although mimivirus amoebal host encodes aaRS, the conservation of aaRS in the mimiviruses’ genomes seems to be associated with an increased viral fitness [38]. The expression of APMV aaRS transcripts can be modulated according to the nutritional status of its host: if mimivirus infection takes place in amoebas cultivated on starvation conditions, a higher mRNA expression of aaRS transcripts is observed. This indicates interplay between nutrient availability sensing of amoeba and the stimulation of the mimivirus aaRS genes as a mechanism related to the circumvention of starvation and maintenance of viral replication in usual levels.

It is intriguing that, even if mimiviruses’ aaRS seem to be true proteins and related to the improvement of viral fitness, there is a trend of aaRS repertoire loss in the taxon. An accordion-like evolution model was recently proposed, suggesting such a pathway as the natural history of many mimivirus genes [39]. Indeed, it is possible to see the gain and loss of a given aaRS and tRNAs (e.g., ArgRS and Leu tRNA), but an overview of aaRS and tRNAs through mimiviruses suggests reduction of aaRS and tRNA classes’ intra- and inter-lineages (Fig 1A). Considering that the occurrence of sequential HGT events involving mimiviruses’ aaRS seems uncertain, this scenario may indicate that the mimiviruses’ ancestor presented a more complete set of aaRS. Also, we can hypothesize that the loss of those genes might be a consequence of viral genome reduction and specialization to a given cell environment and to a more restricted host-range lifestyle (*Acanthamoeba*) (Fig 2). It is noteworthy that this gradual gene loss was evidenced and accelerated experimentally, causing the loss of TrpRS (and other genes) in APMV [40]. Another important piece of this puzzle is the lack of correspondence between mimivirus and *Acanthamoeba* codon/amino acid usages [10]. The requirements of mimiviruses for gene translation are quite different than those of *Acanthamoeba*. However, despite the genomes of mimiviruses of lineages A, B, and C presenting important differences and dissimilarities, the codon and amino acid usages are very similar among the members [10] (Fig 1). But, remarkably, the aaRS gene set present in the known mimiviruses does not match with the viral (or host) codon/amino acid usage demands, providing further evidence that a more complex piece of this puzzle has yet to be found.

### Transfer RNAs in giant viruses—Expanding the translational apparatus

Similar to aaRS, tRNAs are essential molecules for the process of gene translation, being responsible for transporting an amino acid to a template complementary sequence in the molecule of messenger RNA, where the ribosome will further translate the genetic information [41,42]. The tRNAs are largely diffused among the cellular organisms, being the most abundant type of nucleic acid in the cells and constituting up to 10% of all cellular RNAs [39]. Sequences of tRNAs have already been described in some dsDNA viruses, such as members of the family *Myoviridae* [43], *Herpesviridae* [44], and *Phycodnaviridae* [45]. With the discovery of the mimiviruses, the viral tRNA repertoire increased [4].

APMV presents six sequences related to four different tRNAs: leucine (2x TAA and TTG), histidine (CAC), cysteine (TGC), and tryptophan (TGG) [4]. The same tRNAs were found in other mimiviruses of lineage A isolated in Brazil, such as Samba virus, Amazonia virus, Oyster virus, and Kroon virus (except Trp-tRNA) [46], as well as in mimivirus Terra2 [47]. Similar to Kroon virus, Niemeyer virus also has all tRNAs that are found in APMV but Trp-tRNA [15]. It is possible that the evolutionary history of these two viruses differs from that of the other representatives of lineage A, indicating a possible loss of this gene over time. In lineage B, Moumouvirus has sequences related to leucine (TTA), histidine (CAC), and cysteine (TGC), all present in the viruses from lineage A [16]. Considering lineage C, MCV also has three sequences related to tRNA, but only to leucine (TTA and TTG) and tryptophan (TGG) tRNAs [6]. The same tRNAs were identified in the genome of mimivirus Terra1 [47]. By contrast, the mimivirus LBA111 presents all four types of tRNAs found in mimiviruses of lineage A [18]. Similar to viruses from lineage A, it is possible that the ancestor of lineage C viruses had a more complete set of tRNA that was lost during the evolution. If we consider a common ancestor for all group I representatives of the *Mimiviridae* family, the same scenario is reasonable (Fig 1).

In the last years, the family *Mimiviridae* has also expanded the group distantly related to amoeba mimiviruses [48,49]. The analysis of the genome of CroV revealed an even higher range of tRNA-like sequences that had already been identified in their counterparts of group I, with a total of 22 sequences coding for five different tRNAs: leucine (9x TTA), serine (5x TCG), lysine (3x AAA), tyrosine (AAC), and asparagine (AAC), adding some new components in the tRNA set of mimiviruses [5]. Among the algae-infecting mimiviruses, PgV has eight sequences related to tRNA in its genome, coding for leucine (2x TTA e TTG), asparagine (2x AAC), isoleucine (ATA), arginine (AGA), and glutamine (CAA) [19]. OLV presents five tRNAs: leucine (TTG), isoleucine (ATA), tyrosine (TAC), asparagine (AAC), and arginine (AGA) [20] (Table 1). The great diversity of tRNAs coded by mimiviruses of group II is intriguing. Just like for mimiviruses of group I, it is possible that the common ancestor had a more complete set of these molecules that was lost over time. Taking into account the fact that those viruses infect different known hosts (microflagellates and algae), they likely had distinct evolutionary histories and have undergone different selective pressures, which might have contributed to the gain and loss of tRNA genes.

By analyzing the codon/amino acid usage of the mimiviruses and comparing it to the host usage, the hypothesis of multiple events of HGT become even less likely (Fig 2). Among the tRNAs encoded by mimiviruses, leucine (TTA) tRNA is the most common, being present in all of the viruses analyzed so far except for OLV (although it encodes for Leu[TTG]-tRNA), whereas in *Acanthamoeba* sp., it is one of the less frequently occurring tRNAs and is mainly encoded by CTG and CTC [10]. Leu(TAA)-tRNA, which is present in several mimivirus genomes and in multiple copies in some viral genomes, was hypothesized to complement the amoebal tRNA pool and may contribute to accommodating the viral AT-rich codons [10]. The mechanisms of gene expression in the beginning phase of the mimivirus replicative cycle may differ from the mechanisms for gene expression in later phases, and apart from viral RNA transcripts incorporated into mimivirus particles, mimivirus gene expression would first rely primarily on the amoebal machinery and then possibly become increasingly adapted to Mimivirus codon and amino acid usages [10]. In addition, 48% of all mimivirus tRNAs correspond to one of the 10 most frequently used codons in mimiviruses, while 84% of them correspond to one of the 10 least frequently used codons in their hosts [10]. Therefore, such differences suggest that the translational apparatus of the mimiviruses do not come from their currently known natural hosts, which supports the hypothesis that these viruses came from an unknown representative of the fourth TRUC of microbes [30,50,51], although other scenarios cannot be completely ruled out at this point [32,33,52].

### Boosting the viral protein synthesis—Translation factors in mimiviruses

The translation of mRNA into proteins involves three major steps: initiation, elongation, and termination. For these steps to occur, some molecules, named translation factors (TFs), are required. Each step demands specific TFs that are essential for the protein synthesis to occur properly, generically known as initiation factors (IFs), elongation factors (EFs), and release factors (RFs) [53]. Each domain of life presents its own TFs that play similar roles during each step of the process [54]. The viruses do not possess such components, which is why they completely rely on the translational apparatus of their hosts. The discovery of the mimiviruses put this rule to the test, suggesting the existence of a possible fourth TRUC of life [7,50] and a “quasi-autonomous” nature of these giant viruses [55].

The genome of APMV presents sequences homologous to five TFs that are related to all three steps of translation: translation initiation factor 4E, translation initiation factor SUI1, translation initiation factor 4A, translation elongation factor eF-TU, and peptide chain release factor eRF1 [4]. The discovery of these genes, along with aaRS, was a milestone in virology since, until then, no virus was known to harbor sequences related to the translational apparatus, prompting an old debate: whether the viruses are living organisms and if they deserve a special place in the tree of life [4,56,57]. The same TFs were found in the genomes of several other mimiviruses of group I, and some of these genes are also present in the smaller mimiviruses, sequences homologous to IFs being found in the genomes of CroV [5], OLV [20], and PgV [19], but EFs and RFs are absent in these viruses (Table 1). The presence of TFs related to the initial step of translation in representatives of the whole family *Mimiviridae* suggests that these viruses have a weaker dependence on their hosts in the beginning of their replication cycle.

Until now, to the best of our knowledge, there have been no experimental studies about mimiviral initiation or elongation factors, which presents a gap in the biology of mimiviruses. However, work by Saini and Fischer (2007), based on 3-D models and an analysis of the conservation of functionally important residues and motifs, demonstrated that it is possible to derive functional attributes for six APMV ORFans, including initiation factor eIF4E [58]. Regarding the peptide chain RF present in mimiviruses, it was initially suggested it was a class-I RF with a sequence homologous to the RF found in eukaryotes and archaea [4]. The genes that encode eRF1 in APMV and MCV present two stop codons inside the coding region, and for the protein to be accurately synthesized, the viruses must be able to change the reading frame (frameshift recoding event), as well as bypass one of the stop codons (readthrough recoding event), which were considered exclusive features of bacteria [59]. Thus, the analysis of mimivirus RFs revealed a new type of TF that had never been seen before in any known group of organism.

This unique mixture of features of RFs from Eukarya, Archaea, and Bacteria found in mimiviruses raised further questions about their origin. The presence of a completely new type of RF makes its origin by successive events of HGT unlikely, thus supporting the hypothesis that the ancestral mimiviruses constituted a fourth TRUC of life [7,50]. If this scenario is true, we might consider that this ancestor presented a complete apparatus that has been gradually lost over successive speciation events. More studies about the TF of giant viruses will bring new insights about this issue, providing valuable clues to the intriguing mystery that is the origin and evolution of *Megavirales*.

### What comes next? Unraveling the giant viruses’ origin and evolutionary history

Since the discovery of mimiviruses, many theories regarding their origin and evolutionary history have arisen. As soon as the first mimivirus was discovered and its genome analyzed, authors began to hypothesize that this virus stands within the tree (or rhizome [60]) of life [4]. In the following years, the discovery of new giant viruses increased their known pangenome, which supported the initial theories and opened windows for new ones by suggesting that they originated from a fourth TRUC of life [34,50] and also that they probably coexisted with cellular ancestors, evolving mainly through a genome reductive pattern [30,31]. Nevertheless, these theories were readily contested, with some researchers arguing that giant viruses should not be placed in the tree of life and that they came from other small parasitic elements instead of from an extinct branch of life, which had evolved by several HGT events [32,33,56,61,].

Despite many theories, the origin and evolution of mimiviruses remains a breathtaking mystery. It is not yet possible to exclude any specific scenario of giant viruses’ evolution (Fig 2). However, the discovery of new viruses with exceptional genetic content, boosted by the advancement of phylogenomic analysis, provided increasing evidence to support a model wherein the mimivirus ancestor was a more complex organism. Taking this into account, we might speculate that this ancestor possessed a more complete translational-related gene set, which had been constantly losing and gaining genes (mainly through duplication) in accordance with an accordion model of evolution [39]. The *Mimiviridae* ancestor would already be a giant virus, but it would be one with a more independent and generalist lifestyle, able to infect different types of host cells or even interact with them, allowing the gene flow between ancestral lineages. This could have contributed to continuous gene gain and loss over time, shaping the whole viral genome and leading to modern mimiviruses. Considering the new techniques for isolating and discovering giant viruses that are currently implemented [62], we expect to find new mimi- and other giant viruses with genomes that more resemble this ancestor, advancing our understanding of the origin and evolution of this lineage of complex viruses.

## Supporting information

S1 File Arginyl RS alignment. (MAS)

S2 File Asparaginyl RS alignment. (MAS)

S3 File Cisteinyl RS alignment. (MAS)

S4 File Isoleucyl RS alignment. (MAS)

S5 File Metionyl RS alignment. (MAS)

S6 File Tirosyl RS alignment. (MAS)

S7 File Tryptophanyl RS alignment. (MAS)
